# A Case of Opium Cure

**Published:** 1874-03

**Authors:** 


A Case of Opium Cure.—*The Druggist*, of London, says that a young lady who had been long accustomed to the use of opium, applied to an eminent physician to make hypodermic injections of morphia. He commenced by making the injections as desired, of morphia and water. By degrees the quantity of morphia was lessened, without her knowledge, until within a few days nothing but pure water was injected. After each injection she would lapse into a quiet sleep, in the same manner as she had been accustomed to do under the actual use of morphia. This treatment was continued for several months, during which time tonics had been used, to strengthen the system and bring about a healthy condition after being so long a time under the influence of opium. When he considered it safe to do so, he told her plainly that she had not taken a particle of morphia for several months, and was entirely free from its influence. This statement, of course, was received with intense surprise, as well as unbounded joy. The lady is to-day entirely free from any desire for opium.

